# Relationships between obesity, glycemic control, and cardiovascular risk factors: a pooled analysis of cross-sectional data from Spanish patients with type 2 diabetes in the preinsulin stage

**DOI:** 10.1186/1471-2261-14-153

**Published:** 2014-11-01

**Authors:** Luis A Vázquez, Ángel Rodríguez, Javier Salvador, Juan F Ascaso, Helmut Petto, Jesús Reviriego

**Affiliations:** Department of Clinical Research, Lilly, S.A., Avda. de la Industria, 30 28108 Alcobendas, Spain; Clínica de la Universidad de Navarra, Av de Pío XII, 36, 31008 Pamplona, Spain; Hospital Clínico Universitario, University of Valencia, Valencia, Spain; Eli Lilly and Company, Kölblgasse 8-10, 1030 Vienna, Austria

**Keywords:** Cardiovascular disease, Glycosylated hemoglobin A, Prevalence, Obesity, Observational research, Risk factors

## Abstract

**Background:**

Obesity is associated with the onset of type 2 diabetes mellitus (T2D), but reports conflict regarding the association between obesity and macrovascular complications. In this study, we investigated associations between cardiovascular risk factors and body mass index (BMI) and glycemic control in non–insulin-treated patients with T2D.

**Methods:**

Authors gathered cross-sectional data from five observational studies performed in Spain. Generalized logit models were used to analyze the relationship between cardiovascular risk factors (independent variables) and 5 BMI strata (<25 kg/m^2^, 25 to <30 kg/m^2^, 30 to <35 kg/m^2^, 35 to <40 kg/m^2^, ≥40 kg/m^2^) and 5 glycated hemoglobin (HbA1c) strata (≤6.5%, >6.5–7%, >7–8%, >8–9%, >9%) (dependent outcomes).

**Results:**

In total, data from 6442 patients were analyzed. Patients generally had mean values of investigated cardiovascular risk factors outside recommended thresholds. Younger patients had higher BMI, triglyceride levels and HbA1c than their older counterparts. Diastolic blood pressure, systolic blood pressure and triglyceride levels were directly correlated with BMI strata, whereas an inverse correlation was observed between BMI strata and high-density lipoprotein cholesterol (HDL-C) levels, patient age, and duration of T2D. Increased duration of T2D and total cholesterol levels, and decreased HDL-C levels were associated with a higher HbA1c category. BMI and HbA1c levels were not associated with each other.

**Conclusions:**

As insulin-naïve patients with T2D became more obese, cardiovascular risk factors became more pronounced. Higher BMI was associated with younger age and shorter duration of T2D, consistent with the notion that obesity at an early age may be key to the current T2D epidemic. Glycemic control was independent of BMI but associated with abnormal lipid levels. Further efforts should be done to improve modifiable cardiovascular risk factors.

**Electronic supplementary material:**

The online version of this article (doi:10.1186/1471-2261-14-153) contains supplementary material, which is available to authorized users.

## Background

Intricate, heterogeneous sociosanitary, and cultural circumstances are behind the past and projected steady increase in the prevalence of type 2 diabetes mellitus (T2D) [1]. The critical public health priority of diabetes prevention does not imply control of this phenomenon is straightforward. In the absence of effective pharmacotherapy for primary prevention of dysglycemia [2], interventions should be based on weight control, physical activity, and improved quality of diet. However, such interventions are difficult to implement at the population level [3, 4]. Consequently, health systems are increasingly confronted with the task of improving diabetes surveillance and management to reduce the long-term complications of T2D [5]. Robust evidence supports the effectiveness of appropriate glycemic control to prevent microvascular complications in patients with T2D [6–8]. Conversely, there is much less clarity regarding the potential of intensive glycemic control to reduce macrovascular complications of T2D [9–11], although epidemiologic data and meta-analyses have shown a direct relationship between glycemic control and cardiovascular disease [12, 13]. In addition, macrovascular complications have considerable medical relevance because cardiovascular disease is the leading cause of death in people with T2D [8, 14]. Microvascular complications, such as persistent albuminuria, are also important contributors to cardiovascular risk and may be driven by non-traditional risk factors.

Obesity plays a central role in the pathophysiology of both T2D and its macrovascular complications [1, 15]. Nevertheless, some normal-weight individuals have considerable risk of developing T2D and cardiovascular disease because they have a metabolically adverse profile, including hyperinsulinemia, insulin resistance, and hypertriglyceridemia [1, 16]. Thus, a high body mass index (BMI) is not necessary for the occurrence of these conditions, suggesting that the underlying mechanisms of cardiovascular complications of T2D are not straightforward.

Epidemiologic research of cardiovascular risk factors among patients with T2D and different BMI ranges may provide clues as to the relative contribution of obesity to the cardiovascular risk of patients who already have a higher risk of cardiovascular complications because of T2D.

This article reports the results of an analysis of pooled Spanish data from 5 observational studies of patients with T2D during the last decade. The objectives were to investigate the distribution of cardiovascular risk factors among patients across a range of BMI strata, glycated hemoglobin (HbA1c; glycemic control) strata, and age groups.

## Methods

### Design and patients

This report presents a post hoc analysis of cross-sectional demographic and clinical data pooled from the baseline assessments of observational studies of patients with T2D. All patients evaluated in these studies presented within the normal course of care. Only data from patients naïve to insulin therapy and recruited in Spain were included in this analysis.

### Description of source studies

The authors used data from 5 observational studies conducted during the last decade in the primary or secondary (endocrinology or internal medicine) outpatient care settings. One study was multinational, but the current analysis only used data accrued at sites within Spain. The remaining were nationwide studies performed in Spain. The objectives and designs varied between the studies, although all shared a focus on patients with T2D in the preinsulin stage, diagnosed more than 1 year prior to study entry, and collected the same information regarding classical cardiovascular risk factors. Briefly, the studies were:

- Rodríguez et al. [17]: Prospective cohort study of patients with T2D progressing from oral monotherapy to combination therapy. The primary objective related to evaluation of serum lipid profiles. There were 2470 eligible patients from this study.
- Rodríguez et al. [18]: Retrospective and cross-sectional evaluation of patients with T2D naïve to insulin therapy. The primary objective was to assess the quality of healthcare. There were 2264 eligible patients from this study.
- Rodríguez et al. [19]: Cross-sectional evaluation of patients with T2D receiving any therapy. The primary objective was to estimate the prevalence of the metabolic syndrome according to several definitions. Data were available from 1345 patients, but only data from those patients who were not receiving insulin therapy (n = 1066) were selected for this pooled analysis.
- Dilla et al. [20]: Retrospective and cross-sectional evaluation of patients with T2D receiving any therapy. The primary objective was to evaluate the impact of BMI on direct healthcare costs. Data were available from 738 patients, but only data from those patients not receiving insulin therapy (n = 488) were selected for this pooled analysis.
- Costi et al. [21]: Prospective evaluation of patients with T2D starting insulin therapy. The primary objective was to estimate the direct health care costs associated with the first 24 months of insulin therapy. Five European countries participated in this study. Only the baseline data from 178 patients recruited in Spain were eligible for this analysis.

Of the 6466 patients eligible from the included studies, a total of 6442 patients provided sufficient data and were included in the analyses. All provided their informed consent to release information in the respective study they entered. The protocols were in compliance with the Declaration of Helsinki and statutory requirements for observational clinical studies in Spain and were approved by accredited Ethics Committees.

### Data management

The authors collated the following baseline patient data from the source studies: age, sex, height, weight, smoking status, waist circumference, time since T2D diagnosis, type of therapy (lifestyle modifications alone or with oral antidiabetes drugs), HbA1c, fasting blood glucose (FBG), total cholesterol (TC), low-density lipoprotein cholesterol (LDL-C), high-density lipoprotein cholesterol (HDL-C), triglyceride levels, systolic blood pressure (SBP), and diastolic blood pressure (DBP). BMI was taken from studies that provided this information and derived for the remaining patients.

All patients with available data, including patients with protocol violations, were included in the pooled analyses. The rules to define spurious data were: TC or triglycerides >2000 mg/dL, HDL-C >300 mg/dL, SBP/DBP <50/< 30 mmHg, height <100 cm, BMI <10 kg/m^2^ or >100 kg/m^2^, time since T2D diagnosis >100 years, FBG >1000 mg/dL, and HbA1c >50%.

### Data analysis

The data were described as means, standard deviations (SDs) and 95% confidence intervals, or absolute and relative frequencies. Descriptions were stratified by sex, age (<45 years, 45–64 years, 65–75 years, >75 years), BMI (<25 kg/m^2^, 25 to <30 kg/m^2^, 30 to <35 kg/m^2^, 35 to <40 kg/m^2^, ≥40 kg/m^2^), and HbA1c ranges (≤6.5%, >6.5–7%, >7–8%, >8–9%, >9%).

Two cumulative logit models were calculated to analyze the relationship between cardiovascular risk factors (independent variables: TC, LDL-C, HDL-C, triglycerides, SBP, DBP, and current/previous tobacco use) and the ordinal BMI and HbA1c strata described (dependent outcomes). Dummy coding was used to calculate the odds ratios, using the reference categories of <25 kg/m^2^ for BMI and ≤7% (combination of ≤6.5% and >6.5–7% categories) for HbA1c. Both models were reduced using stepwise variable selection at *P* < .05 in the model likelihood ratio test.

The sample size for these analyses was not set a priori, and only the available data from each study were used.

## Results

### Patient characteristics and variation with age

**Figure 1:**
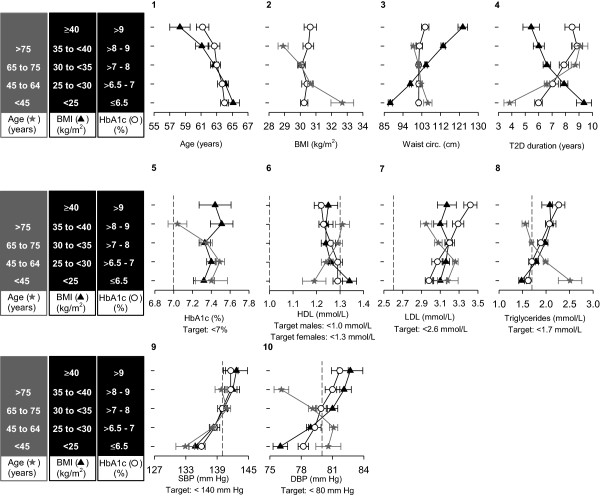
**Classic cardiovascular risk factors and other patient characteristics stratified by age, BMI, and HbA1c ranges.** Values are means and 95% confidence intervals, calculated within the indicated strata of the dependent variables (age, BMI, and HbA1c). The vertical dotted lines in some charts represent target values recommended by the American Diabetes Association [8]. Stars = age; Triangles = BMI; Circles = HbA1c. *Abbreviations:* BMI, body mass index; DBP, diastolic blood pressure; FBG, fasting blood glucose; HbA1c, glycosylated hemoglobin; HDL, high-density lipoprotein; LDL, low-density lipoprotein; SBP, systolic blood pressure; T2D*,* type 2 diabetes.

Table 1 describes the baseline characteristics of the 6442 patients analyzed (Additional file 1: Table S1 provides these data for each study). Overall, slightly less than one-half the patients were women, although this proportion increased with increasing age. The mean (SD) age of patients was 63.2 (10.9) years, and their anthropometric measures indicated that 46.5% were within the range of obesity [overall mean (SD) BMI: 30.3 (5.2) kg/m^2^], predominantly of the central type (mean waist circumference, all: 101.8 cm; women: 99.5 cm, men: 103.7 cm). The mean (SD) duration of T2D was 7.4 (6.7) years, and more than 90% of patients were receiving oral antidiabetes drugs; the remainder was receiving lifestyle change advice only. Mean FBG and, particularly, mean HbA1c were above classical non-individualized recommended targets (mean FBG: 9.0 mmol/L; mean HbA1c: 7.4%). Apart from HDL-C, SBP, and DBP, mean values of the remaining classical cardiovascular risk factors (LDL-C and triglycerides) were above their recommended thresholds. These thresholds are summarized in Figure 1.

**Table 1:** **Characteristics of patients analyzed**

	All patients	Men^a^	Women^a^
Characteristic	(N = 6442)	(N = 3405)	(N = 3036)
Sex: Female [n (%)]	3036 (47.1)	—	—
Current age (years) [mean (SD)]	63.2 (10.9)	62.2 (10.8)	64.3 (11.0)
< 45 [n (%)]	324 (5.0)	186 (5.5)	138 (4.6)
45–64 [n (%)]	3074 (47.7)	1756 (51.6)	1317 (43.4)
65–75 [n (%)]	2209 (34.3)	1082 (31.8)	1127 (37.1)
> 75 [n (%)]	824 (12.8)	374 (11.0)	450 (14.8)
BMI (kg/m^2^) [mean (SD)]	30.3 (5.2)	29.5 (4.4)	31.2 (5.9)
< 25 [n (%)]	769 (11.9)	431 (12.7)	338 (11.1)
25 to <30 [n (%)]	2672 (41.5)	1604 (47.1)	1068 (35.2)
30 to <35 [n (%)]	1979 (30.7)	1010 (29.7)	969 (31.9)
35 to <40 [n (%)]	711 (11.0)	279 (8.2)	431 (14.2)
≥ 40 [n (%)]	311 (4.8)	81 (2.4)	230 (7.6)
Current or previous smoker [n (%)]	2007 (31.2)	1720 (50.5)	287 (9.5)
Waist circumference (cm) [mean (SD)]^b^	101.8 (12.8)	103.7 (12.2)	99.5 (13.1)
Duration of diabetes (years) [mean (SD)]	7.4 (6.7)	7.0 (6.3)	7.8 (7.0)
Patients on oral antidiabetes drugs [n (%)]	6046 (93.9)	3173 (93.2)	2872 (94.6)
HbA1c (%) [mean (SD)]	7.4 (1.5)	7.3 (1.5)	7.4 (1.4)
≤ 6.5 [n (%)]	1911 (29.7)	1055 (31.0)	856 (28.2)
> 6.5–7 [n (%)]	939 (14.6)	490 (14.4)	449 (14.8)
> 7–8 [n (%)]	1664 (25.8)	870 (25.6)	794 (26.2)
> 8–9 [n (%)]	997 (15.5)	493 (14.5)	504 (16.6)
> 9 [n (%)]	767 (11.9)	406 (11.9)	360 (11.9)
FBG (mmol/L) [mean (SD)]	9.0 (2.9)	9.0 (2.9)	9.1 (2.9)
Total cholesterol (mmol/L) [mean (SD)]	5.2 (1.1)	5.1 (1.2)	5.3 (1.1)
HDL-C (mmol/L) [mean (SD)]	1.3 (0.4)	1.2 (0.4)	1.3 (0.4)
LDL-C (mmol/L) [mean (SD)]	3.2 (0.9)	3.1 (0.9)	3.2 (0.9)
Triglycerides (mmol/L) [mean (SD)]	1.9 (1.3)	1.9 (1.5)	1.8 (1.1)
SBP (mmHg) [mean (SD)]	139.1 (17.7)	138.0 (17.1)	140.4 (18.3)
DBP (mmHg) [mean (SD)]	79.7 (10.3)	79.5 (10.3)	80.0 (10.2)

*Abbreviations:* BMI, body mass index; DBP, diastolic blood pressure; FBG, fasting blood glucose; HbA1c: glycosylated hemoglobin; HDL-C, high-density lipoprotein cholesterol; LDL-C, low-density lipoprotein cholesterol; SBP, systolic blood pressure; SD, standard deviation; T2D, type 2 diabetes.

^a^The sex of 1 patient was not recorded.

^b^A total of 3709 patients provided data for this variable.

A greater proportion of men than women was in the overweight range (BMI 25 to <30 kg/m^2^), while women were more frequently in the obese range (BMI ≥30 kg/m^2^), in particular BMI 35 to <40 kg/m^2^. Smoking was more common among men than women (Table 1).There was an inverse relationship between age group and BMI, HbA1c, triglyceride, and DBP levels (Figure 1 stars: charts 2, 5, 8, and 10, respectively). Conversely, as age increased, the duration of T2D also increased, as did HDL-C and SBP levels (Figure 1 stars: charts 4, 6, and 9, respectively).

### BMI and cardiovascular risk factors

Triglyceride, SBP, and DBP levels were directly correlated with BMI strata (Figure 1 triangles: charts 8–10, respectively). Conversely, as BMI increased, HDL-C levels decreased (Figure 1 triangles: chart 6); LDL-C levels did not show strong trends with BMI in either direction (Figure 1 triangles: chart 7). The generalized logit analysis confirmed positive associations between increasing BMI strata and SBP, DBP, and triglyceride levels as significant (*P* < .05 for all). It also indicated an inverse association between increasing BMI strata and HDL-C levels (*P* < .001) and a weaker inverse association between increasing BMI strata and LDL-C levels (*P* < .05).

There was an inverse relationship between BMI strata and both age and duration of T2D (Figure 1 triangles: charts 1 and 4, respectively). The generalized logit analysis confirmed these associations were significant (*P* < .001). HbA1c did not appear to be correlated with BMI strata (Figure 1 triangles: chart 5). The generalized logit analysis also revealed that higher BMI strata was significantly associated with female sex (*P* < .001).

### HbA1c and cardiovascular risk factors

Increased values for the cardiovascular risk factors LDL-C, triglycerides, SBP, and DBP, and decreased levels of HDL-C were related to higher HbA1c strata (Figure 1 circles: charts 7–10 and 6, respectively). In the generalized logit analysis, lower HDL-C (*P* < .001) and higher TC levels (*P* < .001) were associated with higher HbA1c strata. BMI and waist circumference did not show an association with HbA1c (Figure 1 circles: charts 2 and 3, respectively).

There was an inverse relationship between age and HbA1c strata and a positive relationship between duration of T2D and HbA1c strata (Figure 1 circles: charts 1 and 4, respectively). In the generalized logit analysis, duration of T2D was associated with higher HbA1c strata (*P* < .001).

## Discussion

In this population of patients with T2D at the preinsulin stage, most of the modifiable cardiovascular risk factors we investigated showed deviations from recommended goals (see Figure 1 for goals). As BMI strata increased, we found that impairment of most cardiovascular risk factors increased as well, and this was confirmed using generalized logit analysis. Indeed, cardiovascular risk factors were most prominent in patients in the higher BMI strata. This finding is in agreement with that of Gomis et al. [22], who reported an increased frequency of dyslipidemia and hypertension with increasing BMI among 7371 Spanish patients with T2D (*P* < .0001). By contrast, although glycemic control (HbA1c strata) also appeared to be associated with most of the cardiovascular risk factors we considered, the association was significant only for duration of T2D and dyslipidemia.

Noteworthy, BMI strata were also inversely related to age and duration of T2D. This relationship between BMI strata and age is consistent with previously published data [23, 24]**.** Previous epidemiological studies in Spain, performed over the entire age spectrum of the general adult population, had found a direct association between BMI and age up to the fifth or sixth decade [25, 26]. Our patients, all of whom had T2D, had higher BMI values on average than their Spanish population peers [25, 27, 28]. The inverse association between BMI strata and age in the current study may relate to the recognized role that obesity at early ages has in the current T2D epidemic [29–31]. This role would entail a reduction in the age of onset of T2D [29].

In addition, the most recent estimates of T2D prevalence in Spain were considerably high and included a large proportion of undiagnosed cases, which were younger and more obese than previously diagnosed patients [32]. In consequence, in Spain, as in other countries [3], effective prevention and therapeutic strategies, and T2D surveillance specific for overweight or obese youth, are required. It has been shown that obesity in early ages may not permanently increase cardiovascular risk if it is treated successfully [33]. On the other hand, if left unchecked, sustained increases in obesity and T2D prevalence might, in the mid-term, offset the declines in cardiovascular mortality [34] and the positive influences in longevity [35] achieved over recent decades in Western countries.

Although the HbA1c target of below 7.0% was not met by more than one-half the patients in this analysis, 70% of our sample had HbA1c below 8%. Notably, in patients older than 75 years of age, mean HbA1c was close to 7.0%, whereas in all groups of younger patients it was somewhat higher (Figure 1 stars: chart 5). Similarly, Vinagre et al. [36] found that, in Catalonia, patients with T2D aged ≥65 years were more likely to achieve HbA1c ≤7.0% than younger patients. It is likely that the older patients from the studies included in the current analysis were those with a more easily managed hyperglycemia. Glycemic control was related to T2D duration in the current analysis, but this duration was similar in patients aged >75 years and in those aged 65–75 years. A more recent analysis of the Catalan data similarly revealed deterioration in glycemic control with increasing duration of T2D [24]. The major randomized clinical trials that provided evidence supporting intensive glycemic control (ACCORD [37], ADVANCE [38], and VADT [39]) also confirmed the importance of adequate, comprehensive treatment of cardiovascular risk factors to prevent macrovascular complications [10], and this aspect of patient management was insufficiently addressed in the patients of this pooled analysis. When compared with results of a pooled analysis of population-based studies of cardiovascular risk factors performed within the last decade in Spain [40], our patients had similar cholesterol levels, but mean BMI, SBP, and triglyceride levels were higher. Of note, control of cardiovascular risk factors seemed to be slightly better in the systematic evaluation of patients with T2D recently performed in Catalonia, even though about a quarter of those evaluated were receiving insulin therapy and the Catalan population was slightly older than patients in this analysis [36]. We should highlight that the control of glycemia and cardiovascular risk factors might have improved in recent years, because Vinagre et al. [36] gathered information as of 2009, while the present series spanned the last decade.

Our analyses did not show that BMI and waist circumference were associated with HbA1c. This finding is not unexpected, as some individuals with BMI in the obesity range are otherwise metabolically healthy [41]. Indeed, obesity does not seem to always be harmful, and there are some phenotypes in which chronic metabolic inflammation, probably a pivotal condition in obesity and diabetes, is not present. Investigations are therefore underway to identify predictors or biomarkers of healthy versus unhealthy obesity, such as heme oxygenase-1 [42], to allow clinicians to better personalize treatment.

Patients included in our analyses were taking a variety of medications for control of blood pressure and lipid levels, and the blood pressure and lipid levels presented in this manuscript are unadjusted for such medication usage. Although this is consistent with the context of summarizing cardiovascular risk factors in real life, it limits our ability to assess 'underlying' blood pressure or dyslipidemia in isolation.

Strengths of this research include a large sample size and a variety of clinics. In addition, we only included patients naïve to insulin therapy in our analyses to reduce the confounding effect of insulin therapy on weight [43] and cardiovascular risk factors such as hypertension [44, 45]. The limitations include the nature of this post-hoc analysis, performed with heterogeneous observational studies, which were designed with different objectives. As this was not a longitudinal analysis, cardiovascular event rates could not be provided, we instead report a description of cardiovascular risk factors across BMI and HbA1c strata. Moreover, clinical data with known therapeutic or prognostic implications, such as the presence and extent of retinopathy, persistent albuminuria, or non-traditional cardiovascular risk factors/markers were not captured. Regarding body composition, waist circumference data were not available for about 40% of the sample, and data were incomplete to evaluate the ‘healthy’ or ‘unhealthy’ metabolic condition of the obesity seen in patients [46]. Finally, a significant percentage of the patients in this analysis needed a progression in their treatment for hyperglycemia, and this might have introduced a bias towards the inclusion of those patients with worse glycemic control.

## Conclusions

In summary, the results of this pooled analysis in insulin-naïve patients with T2D show that glycemic control is independent of BMI, but this is not the case for other cardiovascular risk factors, such as hypertension and hypertriglyceridemia. Greater effort should be undertaken to reduce cardiovascular risk in patients with T2D, particularly in young, obese individuals, by controlling hypertension and dyslipidemia, as well as blood glucose. Increased emphasis should also be given to treatment strategies for weight control, especially in younger individuals and women.

## Electronic supplementary material

Additional file 1: Table S1: Characteristics of patients analyzed by study. (DOCX 25 KB)

## Abbreviations

BMI: Body mass index
DBP: Diastolic blood pressure
FBG: Fasting blood glucose
HbA1c: Glycated hemoglobin
HDL-C: High-density lipoprotein cholesterol
LDL-C: Low-density lipoprotein cholesterol
SBP: Systolic blood pressure
SD: Standard deviation
T2D: Type 2 diabetes mellitus
TC: Total cholesterol.
