# Supplementary material for: Relationships between obesity, glycemic control, and cardiovascular risk factors: a pooled analysis of cross-sectional data from Spanish patients with type 2 diabetes in the preinsulin stage
Source: BMC Cardiovasc Disord. 2014 Nov 1;14:153. doi: 10.1186/1471-2261-14-153 (PMC4228158; doi:10.1186/1471-2261-14-153)
Supplement: Supplementary file 1 — Additional file 1: Table S1: Characteristics of patients analyzed by study. (DOCX 25 KB) [file 12872_2014_795_MOESM1_ESM.docx]

# Supplementary Table 1 Characteristics of patients analyzed by study

|  | ***Costi et al. 2010 [21]***  ***(N=177)*** | ***Dilla et al. (2012) [20]***  ***(N=486)*** | ***Rodríguez et al. 2010 [17]***  ***(N=2469)*** | ***Rodríguez*** ***Bernardino et al. 2010 [19]***  ***(N=1058)*** | ***Rodríguez et al. 2011 [18]***  ***(N=2252)*** |
| --- | --- | --- | --- | --- | --- |
| **Characteristic** |  |  |  |  |  |
| Sex: Female [n (%)] | 83 (46.9) | 203 (41.8) | 1246 (50.5) | 440 (41.6)^a^ | 1064 (47.2) |
| Current age (years) [mean (SD)] | 65.1 (11.4) | 65.3 (10.9) | 61.1 (10.4) | 64.2 (10.7) | 64.4 (11.1) |
| < 45 [n (%)] | 7 (4.0) | 15 (3.1) | 155 (6.3) | 44 (4.2) | 103 (4.6) |
| 45–64 [n (%)] | 72 (40.7) | 197 (40.5) | 1320 (53.5) | 480 (45.4) | 1005 (44.6) |
| 65–75 [n (%)] | 66 (37.3) | 183 (37.7) | 825 (33.4) | 373 (35.3) | 762 (33.8) |
| > 75 [n (%)] | 31 (17.5) | 91 (18.7) | 163 (6.6) | 158 (14.9) | 381 (16.9) |
| BMI (kg/m^2^) [mean (SD)] | 30.0 (5.7) | 30.1 (4.9) | 30.3 (5.2) | 31.1 (5.3) | 30.1 (5.2) |
| < 25 [n (%)] | 30 (16.9) | 55 (11.3) | 292 (11.8) | 80 (7.6) | 312 (13.9) |
| 25 to < 30 [n (%)] | 61 (34.5) | 216 (44.4) | 1082 (43.8) | 376 (35.5) | 937 (41.6) |
| 30 to < 35 [n (%)] | 48 (27.1) | 143 (29.4) | 723 (29.3) | 396 (37.4) | 669 (29.7) |
| 35 to < 40 [n (%)] | 29 (16.4) | 53 (10.9) | 253 (10.2) | 138 (13.0) | 238 (10.6) |
| ≥ 40 [n (%)] | 9 (5.1) | 19 (3.9) | 119 (4.8) | 68 (6.4) | 96 (4.3) |
| Current or previous smoker [n (%)] | 73 (41.2) | 200 (41.2) | 410 (16.6) | 516 (48.8) | 808 (35.9) |
| Waist circumference (cm) [mean (SD)] | 101.2 (16.8) | 101.6 (12.4) | NA | 104.3 (12.3) | 100.7 (12.5) |
| Duration of diabetes (years) [mean (SD)] | 10.8 (7.1) | 8.6 (6.5) | 8.8 (7.1) | 2.3 (1.1) | 7.5 (6.5) |
| Patients on oral antidiabetes drugs [n (%)] | 171 (96.6) | 446 (91.8) | 2398 (97.1) | 887 (83.8) | 2144 (95.2) |
| HbA1c (%) [mean (SD)] | 9.2 (1.6) | 6.5 (1.0) | 8.1 (1.4) | 7.0 (1.4) | 6.9 (1.2) |
| ≤ 6.5 [n (%)] | 5 (2.8) | 281 (57.8) | 268 (10.9) | 416 (39.3) | 941 (41.8) |
| > 6.5–7 [n (%)] | 3 (1.7) | 94 (19.3) | 254 (10.3) | 179 (16.9) | 409 (18.2) |
| > 7–8 [n (%)] | 26 (14.7) | 76 (15.6) | 768 (31.1) | 240 (22.7) | 554 (24.6) |
| > 8–9 [n (%)] | 57 (32.2) | 24 (4.9) | 644 (26.1) | 89 (8.4) | 183 (8.1) |
| > 9 [n (%)] | 77 (43.5) | 11 (2.3) | 497 (20.1) | 72 (6.8) | 110 (4.9) |
| FBG (mmol/L) [mean (SD)] | 12.0 (3.5) | 7.4 (1.9) | 10.4 (2.9) | 8.5 (2.4) | 7.9 (2.3) |
| Total cholesterol (mmol/L) [mean (SD)] | 4.8 (1.2) | 4.7 (0.9) | 5.5 (1.1) | 5.4 (1.1) | 4.9 (1.0) |
| HDL-C (mmol/L) [mean (SD)] | 1.3 (0.4) | 1.3 (0.3) | 1.2 (0.4) | 1.3 (0.4) | 1.3 (0.4) |
| LDL-C (mmol/L) [mean (SD)] | 2.8 (1.0) | 2.8 (0.8) | 3.4 (0.9) | 3.2 (0.9) | 2.9 (0.9) |
| Triglycerides (mmol/L) [mean (SD)] | 2.0 (1.1) | 1.5 (0.8) | 2.0 (1.5) | 1.9 (1.3) | 1.7 (1.1) |
| Lipid lowering therapy [n (%)] | NA | NA | 947 (41.3)^b^ | 774 61.5^b^ | NA (73)^c^ |
| SBP (mmHg) [mean (SD)] | 136.5 (17.1) | 135.6 (14.4) | 142.4 (18.9) | 139.2 (16.2) | 136.4 (17.3) |
| DBP (mmHg) [mean (SD)] | 78.2 (9.7) | 77.7 (9.9) | 81.8 (10.4) | 80.4 (9.7) | 77.8 (10.1) |
| Antihypertensive therapy [n (%)] | NA | NA | 1240 (54.1)^b^ | 55 4.4^b^ | NA (77)^c^ |

*Abbreviations:* BMI, body mass index; DBP, diastolic blood pressure; FBG, fasting blood glucose; HbA1c: glycosylated hemoglobin; HDL-C, high-density lipoprotein cholesterol; LDL-C, low-density lipoprotein cholesterol; NA, data not available; BP, systolic blood pressure; SD, standard deviation; T2D, type 2 diabetes.

^a^The sex of 1 patient was not recorded.

^b^Proportion of the total population enrolled in the original study from which the current study population was selected.

^c^Population on whom these data are based not available.
